# Prognostic value of circulating regulatory T cell subsets in untreated non-small cell lung cancer patients

**DOI:** 10.1038/srep39247

**Published:** 2016-12-15

**Authors:** Athanasios Kotsakis, Filippos Koinis, Afroditi Katsarou, Marianthi Gioulbasani, Despoina Aggouraki, Nikolaos Kentepozidis, Vassilis Georgoulias, Eleni-Kyriaki Vetsika

**Affiliations:** 1 Laboratory of Translational Oncology, School of Medicine, University of Crete, Voutes, GR-71110, Heraklion, Crete, Greece; 2 Department of Medical Oncology, University Hospital of Heraklion, Voutes, GR-71110, Heraklion, Crete, Greece; 3 Hellenic Society of Immuno-Oncology, 55 Lombardou str., 11474, Athens, Greece; 4 Department of Medical Oncology, 251 Air Force Hospital, Leof. P. Kanellopoulou, 115 25, Athens, Greece

## Abstract

The role of the different circulating regulatory T-cells (Treg) subsets, as well as their correlation with clinical outcome of non-small cell lung cancer (NSCLC) patients is poorly understood. Peripheral blood from 156 stage III/IV chemotherapy-naive NSCLC patients and 31 healthy donors (HD) was analyzed with flow cytometry for the presence and functionality of CD4^+^ Treg subsets (naive, effector and terminal effector). Their frequencies were correlated with the clinical outcome. All CD4^+^ Treg subsets exhibited highly suppressive activity by TGF-β and IL-10 production. The percentages of naive Treg were found elevated in NSCLC patients compared to HD and were associated with poor clinical outcome, whereas the percentage of terminal effector Treg was lower compared to HD and higher levels were correlated with improved clinical response. At baseline, normal levels of naive and effector Treg were associated with longer overall survival (OS) compared to high levels, while the high frequency of the terminal effector Treg was correlated with longer Progression-Free Survival and OS. It is demonstrated, for first time, that particular CD4^+^ Treg subtypes are elevated in NSCLC patients and their levels are associated to the clinical outcome. The blocking of their migration to the tumor site may be an effective therapeutic strategy.

Regulatory T lymphocytes (Treg) play an important role in the homeostasis of immune system, preventing the development of autoimmune diseases. However, in malignant disease, they contribute to the prevalence of immunosuppressive mechanisms by inhibiting the immune response against a variety of cancer cells^1^,^2^,^3^. Treg play a pivotal role in tumor immunology, thereby having an important impact on the outcome of cancer patients^4^,^5^. High levels of peripheral blood Treg prior to therapy have been associated with decreased progression-free survival (PFS) in patients with follicular lymphomas^6^, whereas increased levels of circulating and tumor-infiltrating Tregs, in patients with ovarian and non-small-cell lung cancer (NSCLC), are correlated with worse prognosis and higher risk or recurrence^7^,^8^. Treg exert their suppressive function on effector T cells, namely CD4^+^ and CD8^+^ cells, through several distinct mechanisms including the secretion of inhibitory cytokines such as transforming-growth factor- β (TGF-β) and interleukin-10 (IL-10), the direct contact inhibition via programmed cell death-1 (PD-1), cytotoxic T lymphocyte associated antigen-4 (CTLA-4), indoleamine-pyrrole 2,3-dioxygenase (IDO), T cell immunoglobulin and mucin domain-3 (TIM-3), Lymphocyte-activation gene 3 (LAG-3), and adenosine-prostaglandin E2 (ADO-PGE_2_) pathways and via the secretion of granzymes and other cytolytic molecules^8^,^9^,^10^,^11^.

Multiple markers have been used to better characterize the Tregs, mainly based on their functional characteristics. The transcription factor forkhead box P3 (FoxP3), a crucial intracellular marker, induces peripheral naive T cells to become regulatory T cells with immune suppressive capacity. CD127, the interleukin-7 (IL-7) receptor alpha, plays a vital role in T cell survival and memory phenotype^12^ and its low or no expression (CD127^low/−^) has been proposed as a marker of Tregs^13^,^14^,^15^. Furthermore, the expression of CD152 antigen (CTLA-4)^16^, is fundamental for the immunosuppressive activity of Treg^17^. Nonetheless, there is currently no consensus regarding the appropriate markers that should be used to accurately characterize Treg and their subtypes. Induced Treg (iTreg), which have commonly the CD4^+^CD25^high^ FoxP3^+^CD127^−/low^ phenotype^9^,^18^, are differentiated in the periphery, under the influence of multiple cytokines produced by cells involved in the “inflammation process”, including tumor cells, and are characterized by their high suppressive function^19^,^20^. It has also been proposed that CD4^+^ Treg population could be compartmentalized into “naive”, “effector” and “terminal effector” subtype, bearing unique markers on their surface^21^, in respect to their activation and differentiation stage in the blood circulation. Indeed, based on the expression of CD45RO marker, three Treg subpopulations have been identified^21^,^22^.Naive Treg, defined as CD4^+^CD25^high^CD127^−/low^CD152^-^FoxP3^low^CD45RO^− ^23,24 express high levels of FoxP3 and have suppressive role^25^. They are less sensitive to apoptotic cell death and occur in an earlier stage of differentiation^23^. Effector Treg (CD4^+^CD25^high^CD127^low^CD152^+^FoxP3^+^ CD45RO^+^) represent a short-lived terminally differentiated population, which is divided rapidly and disappears^26^. Terminal effector subtype (CD4^+^CD25^high^CD127^−^CD152^+^FoxP3^+^ CD45RO^+^) is the most efficiently suppressive subtype^27^,^28^, and represents about 20–30% of circulating Tregs^26^.

The data regarding the frequency and the role of circulating Treg subpopulations in NSCLC patients are very limited. Some studies reported significantly higher percentage of CD4^+^CD25^+^FoxP3^+^ Treg in patients with advanced/metastatic NSCLC compared to healthy donors^29^,^30^,^31^,^32^, whereas the high percentage of CD152^+^CD4^+^CD25^high^ FoxP3^+^ Tregs correlates with more advanced stage of disease^29^,^33^. Furthermore, two recent studies demonstrated a prognostic value of peripheral CD4^+^FoxP3^+^Treg in stage I-III NSCLC patients^34^,^35^. However, all studies have focused to a relatively, general population of Treg, which differs from study to study, rather than the quantitative and qualitative assessment of specific Treg subtypes.

There is no consensus regarding the optimal, phenotypic characterization of Treg subtype that could be safely used to better identify patients with more immune suppressive profile, which, eventually, might be of great interest in the era of the rapid evolvement of immunotherapy. Therefore, we sought to identify and investigate the frequency and functional activity of the CD4^+^ Treg subtypes, in NSCLC patients, particularly of three distinct circulating CD4^+^ Treg subtypes (naive, effector and terminal effector). We also correlated their frequency with the disease stage, the histologic subtype and the clinical outcome.

## Results

### Patients and Healthy Donors

Patients’ demographics are presented in Table 1. One hundred fifty-six chemotherapy-naive patients were enrolled in the study. All patients were diagnosed with inoperable, locally advanced (stage III) or metastatic (Stage IV) NSCLC and were treated with 4–6 cycles of platinum-based chemotherapy regimens with or without bevacizumab (12.2% and 87.8%, respectively). The median age was 62 years, 82.1% were men, 57.6% had an adenocarcinoma, and 82.1% had stage IV disease. Eighty-eight (56.4%) patients were evaluable for assessment of clinical outcome, while 60 patients were not clinically evaluated because of early death or treatment discontinuation for medical reasons. Eight patients refused any systemic treatment.

### Percentage of circulating CD4^+^Treg subpopulations in NSCLC patients

**Figure 1:**
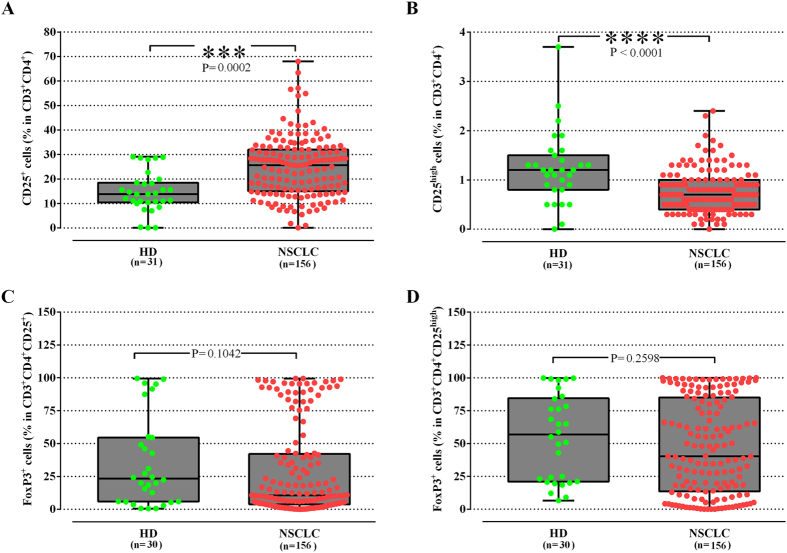
NSCLC patients show a significant increase in CD4^+^CD25^+^ T cells compared to healthy donors. (**A**) The percentage of CD4^+^CD25^+^, (**B**) CD4^+^CD25^high^, (**C**) CD4^+^CD25^+^FoxP3^+^ and (**D**) CD4^+^CD25^+^FoxP3^high^ Tregs in the peripheral blood of 156 NSCLC patients was determined by flow cytometry and compared with 31 healthy donors (HD). The study group did not show significantly different percentage of CD4^+^CD25^+^FoxP3^+^ and CD4^+^CD25^+^FoxP3^high^ Tregs than HD. Each point corresponds to an individual patient (red circle) or HD (green circle). The medians, 75 percentile (box) and max and min (whiskers) are represented. Groups were compared by nonparametric Kolmogorov-Smirnov test.

The percentage of CD4^+^CD25^+^ Treg cells was significantly increased in NSCLC patients (24.81 ± 1%) compared to HD (14.67 ± 1.5%; p = 0.0002; Fig. 1A). Their expression remained significantly elevated compared to control group, regardless the tumor histology or the stage of disease (Supplementary Table S1). However, there was no significant difference of the percentage of Treg between the different clinical stages (stage III versus IV) or histologies (squamous versus adenocarcinoma). In contrast, the percentage of another commonly used subpopulation, the CD4^+^CD25^high^, was significantly lower in patients compared to HD (0.75 ± 0.03% versus 1.23 ± 0.1%, respectively; p < 0.0001; Fig. 1B), irrespectively of the stage and histology (Supplementary Table S1).

The proportion of CD4^+^CD25^+^ and CD4^+^CD25^high^Treg expressing the transcription factor FoxP3^+^ was, subsequently, analyzed. There was no significant difference between the percentages of CD4^+^CD25^+^FoxP3^+^ or CD4^+^CD25^high^FoxP3^+^ Treg in NSCLC patients and HDs (29.04 ± 2.8% versus 35.46 ± 6.2%; p = 0.1 and 47.09 ± 2.8% versus 54.81 ± 5.9%; p = 0.3, respectively, Fig. 1C,D). The frequencies of both subpopulations did not differ between the tumor stages (Supplementary Table S2). Patients with adenocarcinoma had a significant lower percentage of CD4^+^CD25^+^FoxP3^+^ (26.22 ± 3.53%) and a numerical lower percentage of CD4^+^CD25^high^FoxP3^+^ (41.89 ± 3.74%) than HD (35.46 ± 6.23, p = 0.03 and 54.81 ± 5.9%, p = 0.13,respectively), whereas patients with squamous cell carcinoma had significantly higher frequencies of CD4^+^CD25^high^FoxP3^+^compared to patients with adenocarcinoma (57.44 ± 4.83% versus 41.89 ± 3.74%, p = 0.006; Supplementary Table S2).

### Detection of circulating naive, effector and terminal effector Treg in NSCLC patients

**Figure 2:**
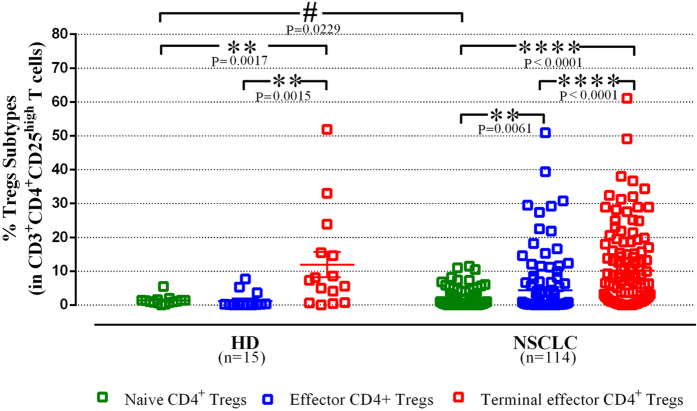
Percentage of CD4^+^ Treg subtypes in NSCLC patients and healthy donors. The percentages of the naive Treg (green open square) were statistically increased in NSCLC patients compared to healthy donors (HD). In contrast, there was no significant difference between NSCLC patients and healthy donors in the effector (blue open square) and terminal effector (red open square) Treg subtypes. The percentage of terminal effector Treg was the most dominant subtype compared to the other two, in NSCLC patients. Each point corresponds to an individual patient or healthy donors. The p values are determined by nonparametric Kolmogorov-Smirnov test (between HD and NSCLC patients) and Wilcoxon matched-paired signed rank test between different subtypes.

Treg were further investigated according to their stage of activation and differentiation. Three subtypes could be identified: naive (CD25^high^CD127^−/low^CD152^-^FoxP3^low^CD45RO^−^), effector (CD25^high^CD127^low^CD152^+^FoxP3^+^CD45RO^+^) and terminal effector (CD25^high^CD127^−^CD152^+^FoxP3^+^CD45RO^+^) Treg. The percentages of naive CD4^+^ Treg were increased in NSCLC patients compared to HD (1.6 ± 0.2% vs 1.33 ± 0.3%; p = 0.02). In contrast, no difference in the frequencies of effector Treg was found (1.21 ± 0.6% vs 4.35 ± 0.83%; p = 0.24) between the HD and NSCLC patients. Concerning the percentage of circulating terminal effector Treg in NSCLC patients, even though they were numerically lower compared to HD, no significant difference was observed (10.22 ± 1.1% vs 11.95 ± 3.74%; p = 0.68; Fig. 2). Conversely, the terminal effector subtype was the most expanded subtype in comparison with naive and effector Treg subtypes (p < 0.0001; Fig. 2) in both HD and patients.

Following the above observation, we tested whether pathological type or clinical stage influenced the presence of these three subsets. The analysis of Treg based on histologic subtype revealed that naive Treg were decreased in adenocarcinoma compared to HD (1.22 ± 0.26% vs 1.33 ± 0.33; p = 0.01), while no difference was observed between squamous and other NSCLC subtypes and HD (Supplementary Table S3). There was no difference regarding the frequency of effector Treg compared to HD, in the different NSCLC subgroups (histology and stage). Finally, the percentage of terminal effector Treg in adenocarcinoma was significant lower compared to squamous histology (p = 0.0008; Supplementary Table S3). The percentages of naive and terminal effector Treg subtypes were not different between the variant clinical stages. However, patients with stage IV NSCLC had significantly higher percentage (5.04 ± 0.97%) of effector Treg compared to stage III (1.15 ± 0.95%, p = 0.03) disease (Supplementary Table S3).

### Functionality of the distinct Treg subtypes in NSCLC

**Figure 3:**
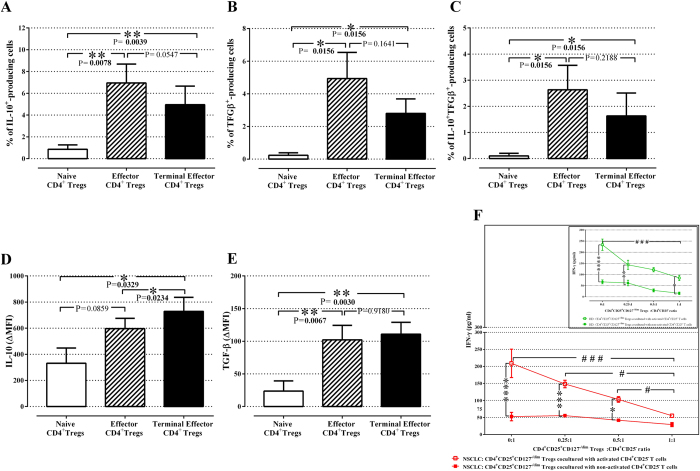
Functionality of CD4^+^ Treg subtypes in NSCLC patients. Percentages of (**A**) IL-10^+^, (**B**) TGFβ^+^and (**C**) TGFβ^+^ IL-10^+^-producing naive, effector and terminal effector Treg from NSCLC patients. Percentages indicated in the plots represent the percentages of phenotypic marker expression in the Treg subtypes. The data are represented as the mean ± SEM and the *P* values are determined by the Wilcoxon matched-paired signed rank test. Intracellular levels of (**D**) IL-10 and (**E**) TGF-β in the three Treg subtypes. Bars represent the average ΔMFI (median fluorescence intensity corresponded to unstained control subtracted from median fluorescence intensity of specific Ab). Data are presented as mean ± SEM and the *P* values are determined by the Wilcoxon matched-paired signed rank test. (**F**) Inhibitory effect of Treg on CD4^+^ T cell in NSCLC patients and in HD (inset figure). IFN-γ levels in the supernatant from co-cultures of activated (NSCLC: red open square; HD: green open square) or non-activated (NSCLC: red closed square; HD: green closed square) CD4^+^T cells with Treg isolated from NSCLC patients and HD detected by ELISA. The experiments were performed in duplicates. The data shown are of five independent experiments and represented as mean values ± SEM and the p values are determined by Friedman test. CD4^+^ Treg subtypes; naive (CD25^high^CD127^−/low^CD152^−^FoxP3^low^CD45RO^−^); effector (CD25^high^CD127^low^CD152^+^FoxP3^+^ CD45RO^+^) and terminal effector (CD25^high^CD127^−^CD152^+^ FoxP3^+^CD45RO^+^).

In order to investigate the suppressive function of different CD4^+^ Treg subtypes in NSCLC patients, we assessed the secretion of the immunosuppressive cytokines TGF-β and IL-10 by these cells using flow cytometry analysis (see Supplementary Fig. S2). It was found that all subtypes secreted both TGF-β and IL-10. The percentage of naive Treg producing both IL-10 and TGF-β was significantly lower compared to effector and terminal effector CD4^+^ Treg producing IL-10 and TGF-β (Fig. 3A–C). Moreover, the terminal effector Treg had the highest expression of IL-10, as determined by the median fluorescence intensity (ΔMFI), compared to naive (p = 0.03) and effector (p = 0.02) Treg. Although the percentage of effectors and terminal effectors Treg producing IL-10 were numerically comparable, the levels of the IL-10 expression were significantly higher in terminal effector Treg than in effector Treg (p = 0.02; Fig. 3A,D). Both effector and terminal effector Treg expressed higher amounts of TGF-β ( = 0.007, p = 0.003; respectively Fig. 3C) compared to naive Treg (Fig. 3D,E). Finally, the expression levels of IL-10 in all subtypes were higher compared to TGF-β levels (Fig. 3D,E).

### Suppressive activity of Treg (CD4^+^CD25^+^CD127^−/dim^) in NSCLC cancer patients and healthy donors

In order to assess the suppressive ability of the above mentioned Treg subsets in NSCLC patients and HDs, isolated enriched Treg of all stages of activation and differentiation (CD127^−/low^CD25^+^CD4^+^) were co-cultured with activated CD4^+^ T-cells and culture supernatants were tested for IFNγ production. Figure 3F clearly indicates that Treg significantly decreased IFNγ-production by activated CD4^+^ T-cells in a dose-dependent manner in NSCLC patients. Isolated CD4^+^ Treg (CD127^−/low^CD25^+^CD4^+^) from HDs showed an equivalent suppressive capacity (Fig. 3F, inset).

### Correlation of the different subtypes of circulating Treg with the clinical outcome

Patients with stage IV disease experiencing disease progression (PD) during front-line chemotherapy had significantly increased percentages of baseline naive Treg (CD25^high^CD127^−/low^CD152^-^FoxP3^low^CD45RO^−^; 3.17 ± 0.58%) compared to those who achieved disease control (DC) (0.81 ± 0.33%; p = 0.003). In contrast, baseline high levels of terminal effector Treg were correlated with improved clinical response (PD versus DC: 7.48 ± 1.27% versus 14.08 ± 2.74%; p = 0.04). Finally, effector Treg were not correlated with the response to treatment (PD versus DC: 6.02 ± 2.1% versus 1.52 ± 0.7%; p = 0.77, Table 2).

**Figure 4:**
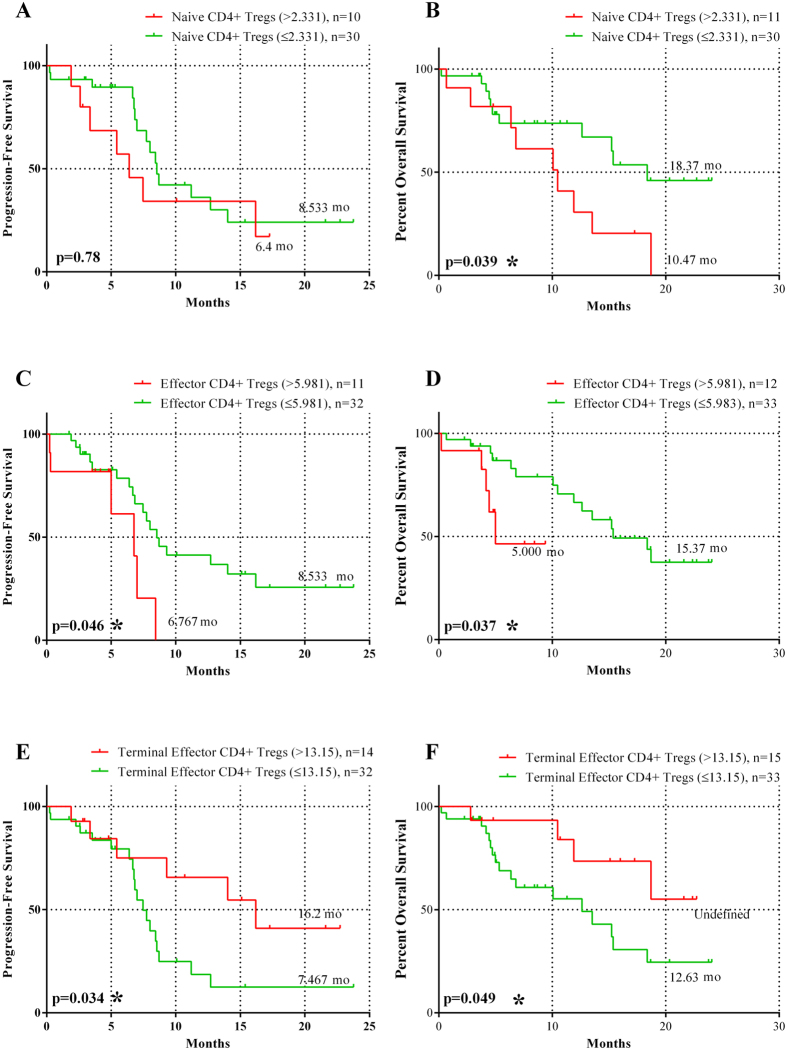
Prognostic significance of Naive, Effector and Terminal Effector CD4^+^ Treg in NSCLC patients. Kaplan-Meier survival analysis of patients divided according to the percentages of naive, effector and terminal effector CD4^+^ Treg. Normal percentages of Naive and Effector CD4^+^ Treg were associated with improved (**A**,**C**) progression-free survival and (**B**,**D**) overall survival. In contrast, normal percentage of Terminal Effector CD4^+^ Treg was associated with both (**A**) shorter PFS (P = 0.0341) and (**B**) OS (P = 0.0492); dashed line: below 95%, solid line: above 95% of normal control.

Assuming increased levels of Treg subtypes those that were over the 95% percentile of the HD, the stage IV patients, who received chemotherapy, were dichotomised to those with Treg percentage above and those within the normal range. The detection of naive Treg in patients with normal or high levels, at baseline, did not altered the progression-free survival [(PFS) 6.4 vs 8.5 months; p = 0.79; Fig. 4A]. Yet, the patients with normal naive Treg levels achieved a significantly longer overall survival [(OS); 18.37 versus 40.47 months; p = 0.039; Fig. 4B] in comparison to patients with high levels. Similarly, the subgroup of patients with high percentage of effector Treg at baseline had shorter PFS (6.8 vs 8.53 months; p = 0.046) and OS (5 months vs 15.37 months; p = 0.037; Fig. 4C,D) compared to those within normal range. In contrast, high frequency of the terminal effector Treg at baseline was correlated with longer PFS (16.2 vs 7.5 months; p = 0.03) and OS (undefined vs 12.63 months, respectively; p = 0.049; Fig. 4E,F) compared to low frequency.

Univariate analysis revealed that high baseline expression of naive Treg was significantly associated with decreased OS (p = 0.046). Low expression of effector Treg was significantly correlated with increased PFS (p = 0.032) and OS (p = 0.049), whereas low expression of terminal effector Treg was significantly associated with decreased PFS (p = 0.039) and OS (p = 0.05; Table 3A). Multivariate analysis revealed that high levels of terminal effector Treg is an independent factor associated with increased PFS (HR = 3.47; 95% CI: 1.005–11.946, p = 0.049) and OS (HR = 7.417; 95% CI: 1.055–52.151, p = 0.044). In contrast, high levels of naive Treg emerged as an independent factor associated with decreased OS (HR = 8.632; 95% CI: 2.226–33.468, p = 0.002; Table 3B).

## Discussion

Although regulatory T-cells play a critical role in the maintenance of immunological homeostasis and self-tolerance, in cancer patients, these cells contribute to the establishment of immune suppressive conditions. Several groups have studied different Treg subpopulations, both in tumor microenvironment and in peripheral blood and they have investigated their role in solid tumors. However, there is no consensus regarding the phenotypic characterization of Treg. In NSCLC, little is known about the expression of different circulating subtypes of Treg and their contribution in the tumor development, progression, and eventually, the disease clinical outcome. In the current study, the expression of different subtypes of circulating Tregs, in newly diagnosed NSCLC patients before the administration of any systemic or local treatment, was investigated. We further analyzed the different subtypes of circulating Treg according to the expression of markers associated with their suppressive function, such as FoxP3, CTLA-4 and IL-7R alpha. Finally, we correlated the levels of the distinct subtypes with the clinical outcome of the patients.

Several reports in NSCLC patients have described Tregs as CD4^+^CD25^+ ^36 and have shown that this population is elevated in comparison to healthy donors^31^,^37^,^38^.Therefore, we used this general phenotype as a starting point. The present data showed a significant 1.7-fold increase of CD4^+^CD25^+^ Treg cells compared to healthy donors, in accordance with previous reports^31^. In contrast, no difference was detected in the percentage of CD4^+^CD25^+^FoxP3^+^ Treg between the whole NSCLC population and HD, also, in agreement with a previous study^39^. However, other groups have shown increased levels of this particular subpopulation in NSCLC patients compared to healthy controls^34^,^40^. These contradictive results may be explained by the fact that in our study the HD and NSCLC patients were age-matched, whereas in the other studies the median age of the healthy donors was lower compared to the patients^40^. Pan and colleagues have shown that the increased number of Treg and the overexpression of FoxP3 gene are correlated to aging, as healthy elderly had a higher proportion of peripheral Treg compared with younger healthy donors^41^.

Another subpopulation of Treg (CD4^+^CD25^high^ cells), that has been proposed having even higher suppressive properties^27^, was found in significantly lower levels in NSCLC patients compared to the healthy donors (p < 0.0001), regardless the clinical stage or the histology. These results are contradictive to other studies in similar patient population^42^,^43^. We further investigated whether FoxP3 could be a likely mechanism of function of these highly suppressive Treg, as it has been suggested in studies in other tumor types^44^. In the current study, no statistically significant differences in the percentage of CD4^+^CD25^high^FoxP3^+^ Treg subtype between NSCLC patients and HDs were observed. However, an important finding in the present study was the fact that in the adenocarcinoma patients, the frequency of this subpopulation was significantly decreased compared to HD and those with squamous cell carcinoma. The fact that adenocarcinoma patients were the majority of the enrolled patients, could be an explanation for the discordance occurred in the current study compared to the corresponding data from other studies. Moreover, the higher expression of this subpopulation in squamous cell carcinoma may imply that this histologic type might be more immunogenic, resulting eventually, to an increased expression of suppressive cells. The addition of other markers involved in the suppressive function of Treg, such as CD152 and CD127, allowed the identification of a more homogeneous cell population in different carcinomas^45^.

CD4^+^CD25^high^FoxP3^+^ Treg are a heterogeneous population in different stages of activation and differentiation. Thus, naive, effector and terminal effector Treg, which can be detected in the circulation, exert their suppressive function with distinct mechanisms. In the present study, for the first time, the three subtypes of Treg were identified and quantified in NSCLC patients. Increased percentages of naive Treg were revealed compared to healthy donors (p = 0.02); however, the percentage of naive Treg was significantly lower in patients with adenocarcinoma than in healthy controls (p = 0.02), implying that naive Treg were mainly increased in squamous cell carcinoma (Supplementary Table S3). It was also observed that the increased percentage of naive Treg, at baseline, was correlated with disease progression at the first treatment evaluation (Table 2). Moreover, naive Treg were associated with resistance to chemotherapy and worse clinical outcome in terms of PFS and OS (Fig. 4A,B). This observation may be related to the fact that naive Treg are less sensitive to apoptotic cell death^23^,^25^ and, therefore, based on their immunosuppressive properties, may strongly contribute to the down-regulation of the immune response against tumor cells.

Effector Treg are predominant among tumor-infiltrating FoxP3^+^ T cells with suppressive function, while their frequency is higher in TILs compared to peripheral blood^46^. Indeed, the current study confirmed that there was no difference regarding the percentage of effector Treg between NSCLC patients and HD, as well as between the distinct histology subtypes (Fig. 2; Supplementary Table S3). However, it was shown that the percentage of effector Treg was significantly higher in stage IV patients compared to stage III (p = 0.03). Moreover, the majority of these cells produced IL-10 and TGF-β, in contrast to the other two subtypes, naive and terminal effector cells (Fig. 3A–C). In addition, no correlation was observed in the percentage of the effector Treg at baseline and response to 1^st^ line treatment (Table 2). However, the group of patients with high percentage of effector Treg at baseline had shorter PFS and OS compared to the group with low percentage (Fig. 4C,D). It is known, from preclinical studies, that effector Treg could efficiently suppress conventional effector T-cell responses *in vitro*, which possibly reflect their effect in clinical outcome^47^.

Terminal effector Treg are part of the effector Treg compartment of which they seem to constitute a terminally differentiated subset^26^ with a more efficient and rapid suppressive function compared to other subtypes^28^. In this study, no significant difference was observed in the percentage of terminal effector Tregs between patients and HD (Fig. 2). However, a significant increase was observed in the percentages of terminal effector Treg in patients with squamous cell carcinoma or other NSCLC subtypes compared to adenocarcinoma patients. Moreover, this subset expressed higher levels of immunosuppressive cytokines (IL-10 and TGF-β), compared to the other subtypes (Fig. 3D,E). In addition, a correlation between increased circulating terminal effector Treg percentages and better clinical outcome in terms of higher response rate, longer PFS and OS was observed (Table 2 and Fig. 3D,E). One possible explanation might be that the increased levels of circulating terminal effector Treg may reflect their decreased levels in tumor site^48^. As these cells inhibit tumor immune reactions by direct cell-to-cell contact, the increased circulating terminal effector Treg population in the blood may not influence the immune response against cancer cells. Consequently, high levels of terminal effector Treg in the blood stream may lead to a significant survival advantage.

On the other hand, chronic inflammation is considered as one of the major risk factors for the development of lung cancer. Therefore, the presence of high levels of circulating terminal effector Treg may contribute to dampen these local inflammatory responses^49^. It can be hypothesized that the increased levels of circulating terminal effector Treg is the result of this highly immunogenic tumor type. It has been suggested that tumors with strong immune response have better clinical outcome compared to those with no immunological response. Several studies have also suggested the positive prognostic role of Treg in patients with different types of cancer such as in colorectal cancer; indeed, high levels of CD45RO^+^ and FoxP3^+^ infiltrating Treg have been associated with improved survival and could be emerged as independent prognostic factor for longer OS^50^,^51^,^52^. In addition, high levels of CD25^+^FoxP3^+^ tumor-infiltrating T-cells were associated with favorable prognosis in triple negative breast cancer patients^53^. Finally, high percentages of circulating Tregs have been correlated with longer overall survival compared to low levels in patients with oropharyngeal squamous cell carcinoma^54^. Taking all the above mentioned data into consideration, future studies should be focused on better understanding of the precise role of the terminal effector Treg in cancer patients, both in circulation and tumor microenvironment.

The present study also demonstrated the independent predictive and prognostic value of distinct CD4^+^ Treg subtypes. Normal levels of naive and effectorCD4^+^ Treg, at baseline, were associated with better patients’ PFS and OS compared to patients with high levels, whereas normal levels of terminal effector CD4^+^ Treg were correlated with worse clinical outcome. Therefore, the functional heterogeneity of CD4^+^ Treg (naive, effector and terminal effector) should be taken into account, when the role of CD4^+^ Treg in prognosis of NSCLC patients is proposed. The limitation of this study is that, due to insufficient biological material, we were unable to isolate the distinct Treg subtypes in order to evaluate their suppressive function *in vitro.* Despite this fact, we performed co-culture experiments using an enriched isolated Treg population (CD4^+^CD25^+^CD127^−/dim^) from both NSCLC patients and HDs and proved that this enriched population had suppressive capacity, since they could reduce the production of IFNγ by activated CD4^+^ T cells (Fig. 3F).

To conclude, the data presented in the current study demonstrate that the presence of naive and effector CD4^+^ Treg are clearly correlated with poor clinical outcome, while high expression of terminal effector cells correlates with better clinical outcome in patients with metastatic NSCLC. The expression of these subpopulations might be exploited as a potential predictive, as well as prognostic biomarker in future studies, especially in the current era of immunotherapy. Moreover, better understanding of the role of these subpopulations in the carcinogenesis and tumor development will potentially allow for superior selection and stratification of patients in Treg-targeting therapies, which in turn contribute to better clinical outcome. Finally, based on the present findings, future immunotherapies need to take into consideration, the distinct Treg subsets, instead of a general population, in order to enhance their effectiveness in the NSCLC.

## Materials and Methods

### Patients and Healthy Donors’ Samples

Peripheral blood in EDTA (BD Biosciences, Europe) was obtained from 156 chemotherapy-naive NSCLC patients at the time of diagnosis, and before the administration of any treatment and 31 age- and sex-matched healthy volunteers [23 males and 8 females; age 64 ± 3 years; healthy blood donors; (HD)]. All patients were older than 18 years and had not received any immunosuppressive drugs or granulocyte-colony stimulating factor (G-CSF) prior to immune testing. Blood samples from HD were used as controls. The methods were carried out in accordance with relevant guidelines. The study complied with the Ethical Principles for Medical Research Involving Human Subjects according to the World Medical Association Declaration of Helsinki and was approved by the local ethics and scientific committees of the University Hospital of Heraklion (Greece), No.17869–16/12/2014. All patients and HDs provided a written informed consent in order to participate in the study.

### Cell isolation and Flow cytometry for immunophenotypic analysis of cells

Peripheral blood (6 ml) was centrifuged and the plasma was removed and stored at −80 °C. For flow cytometry analysis, blood samples underwent red blood cell lysis using Red Blood Cell (RBC) lysing buffer according to the manufacturer recommendations (BD Biosciences; USA). Briefly, 5 ml EDTA-treated whole blood was added into a tube containing 45 ml RBC lysing buffer at room temperature. Following 20 min incubation at room temperature, the tubes were centrifuged at 500 g for 5 min. The supernatant was discarded and the white blood cell pellet was washed twice with 15 ml flow buffer (1% FCS, 0.01% NaN_3_ in PBS; Sigma, USA) and cells were then re-suspended in flow buffer (1 × 10^7^/ml) for immunophenotypic analysis.

Fluorescence-active cell sorting (FACS) analysis was performed on freshly isolated cells. White blood cells were stained for expression of surface markers using anti-human monoclonal antibodies conjugated to fluorochrome against different molecules: anti-CD4-V500; anti- CD3-PE-CF594; anti-CD25-PE-Cy7; anti-CD127-V450 and anti-CD45RO-Alexa700 (BD Biosciences, USA). Staining was performed for 30 min, on ice in dark. For intracellular staining, the cells were fixed and permeabilized using FoxP3 Buffer set (BD Biosciences) according to manufacturers’ instructions and stained for FoxP3-FITC, CTLA-4-PE-Cy5, TGFβ and IL-10 for 1 h on ice in dark. After washing, cells were re-suspended in 0.5 ml FACS buffer and a multicolour analysis was performed using a BD LSR II Flow Cytometer (BD Biosciences). Analysis of FACS data was done using FACS Diva Software (BD Biosciences). For T-cell subset, the acquisition and analysis gates were restricted to the lymphocyte population. Each measurement contained 10^6^single cells. The gating strategy for Treg populations (A) and subtypes (B) is shown in Supplementary Fig. S1. The expression levels of IL-10 and TGFβ- are reported as ΔMFI (median fluorescence intensity of the specific antibody minus the corresponding median fluorescence intensity of the negative control). Unstained cells were used as negative control.

### Isolation of Treg and *in vitro* suppression assay

Peripheral blood mononuclear cells (PBMC) were obtained after Ficoll-Hypaque density (Sigma, UK) density centrifugation of peripheral blood (50 ml in EDTA) from five treatment-naive patients. PBMCs were washed in AIM-V medium and immediately used for Treg isolation by magnetic separation using immunomagnetic beads and the autoMACS system (MiltenyBiotec GmbH, Germany).

Immunomagnetically purified CD4^+^ CD25^+^CD127^−/dim^Treg and CD4^+^ CD25^−^ T cells were obtained from PBMCs using the CD4^+^CD25^+^CD127^−/dim^ Treg isolation kit and the CD25 and CD4 microbeads (Milteny Biotec GmbH, Germany), according to the manufacturer’s instructions. Patients’ and HDs’ PBMCs were incubated with a mix of biotin-conjugated antibodies (CD8, CD19, CD123, and CD127) for 5 minutes at 4° to 8 °C followed by addition of anti-biotin microbeads and further incubation for 10 minutes 4° to 8 °C. After incubation, cells were resuspended in MACS buffer, and CD127^dim/−^ CD4^+^ Tregs were isolated using negative selection. CD25 microbeads II were then added in the CD127^dim/−^ CD4^+^ cell suspension for 15 minutes at 4° to 8 °C, and finally the CD4^+^ CD25^+^CD127^−/dim^Treg were isolated using positive selection. CD4^+^CD25^-^T cells were isolated from the PBMCs of the corresponding patients and HDs also using antibody-coated magnetic beads against CD4 and CD25 by CD25 negative selection followed by CD4 positive selection (Miltenyi Biotec GmbH, Germany). The isolated cells were identified using flow cytometry and their purity was greater than 90%. Viability of isolated cells was measured using 0.1% trypan blue.

CD4^+^CD25^−^ T (responders) were seeded in 96-well plates at 1 × 10^5^cells/well and were stimulated using Dynabeads Human T-Activator CD3/CD28 beads (Invitrogen, Carlsbad, CA) according to manufacturer’s instructions. CD4^+^CD25^+^CD127^−/dim^ Treg (effector cells)were co-cultured with autologous activated or non-activated responder cells (CD4^+^CD25^−^ T) at a ratio of 1:1, 0.5:1 and 0.25:1 (effector: responder cell ratio). For each patient, HD and assay run, controls included T cells cultured alone (0% suppression) with and without stimulation. After a 48-hour co-culture, the supernatant was obtained by centrifugation at 500 g for 5 min and the IFN-γ levels were detected using Enzyme-linked immunosorbent assay (ELISA) according to the manufacturer’s instructions (R&D Systems, Minneapolis, MN).

### Statistical analysis

Statistical analysis was performed using GraphPad Prism version 6.0 (GraphPad Institute Inc, USA). Data are presented as mean ± SEM. Differences between groups were determined using the Kolmogorov-Smirnov (KS) non-parametric test, Unpaired T test, Fieldman test and Wilcoxon matched-pairs signed rank test, as stated. High expression of Treg was defined as the percentage of the cells above the 95% percentile of the controls. Median OS and PFS were estimated using the Kaplan-Meier method with groups compared using the log-rank test. OS was defined as the time from the study enrolment to death. PFS was defined as the time between the enrolment and the first date of first observation of clinical progression or death. Univariate and multivariate Cox regression hazards model were performed using the SPPS Statistics 20 software (SPSS Inc, USA). All patients included in the univariate and multivariate Cox regression analysis had stage IV disease and received chemotherapy regimens. Differences and associations were considered significant when p < 0.05.

## Additional Information

**How to cite this article**: Kotsakis, A. *et al*. Prognostic value of circulating regulatory T cell subsets in untreated non-small cell lung cancer patients. *Sci. Rep.* **6**, 39247; doi: 10.1038/srep39247 (2016).

**Publisher’s note:** Springer Nature remains neutral with regard to jurisdictional claims in published maps and institutional affiliations.

## Supplementary Material

Supplementary Information

## Figures and Tables

**Table 1:** Patients’ demographics.

	Patients (n = 156)	%
Median age		
years (range)	62 (47–89)	
Sex		
Male	128	82.1
Female	28	17.9
Histology		
Adenocarcinoma	90	57.6
Squamous	49	31.4
Other types	17	11
Stage		
ΙΙΙΑ/Β (non eligible for radiation)	28	17.9
IV	128	82.1
Treatment regimens		
Platinum-based	131	84
Taxane-based+bevacizumab	15	9.6
Taxane (single agent)	6	3.8
Taxane(single agnet)+bevacizumab	4	2.6
Response to therapy		
PR	25	16.1
SD	39	25
PD	24	15.4
NE	68	43.5

NE: Non-Evaluated, PR: partial response, SD: stable disease, PD: progressive disease.

**Table 2:** Correlation of the percentage of Naive, Effector and Terminal Effector CD4^+^ Tregs with response to 1^st^ line treatment.

Response to 1^st^ line treatment	% of Naïve CD4^+^ Tregs Mean ± SEM	P value	% of Effector CD4^+^ Tregs Mean ± SEM	P value	% of Terminal Effector CD4^+^ Tregs Mean ± SEM	P value
PD (n = 30)	3.17 ± 0.58**	0.003	6.02 ± 2.2	0.77	7.48 ± 1.3*	0.04
Non-PD (n = 18)	0.81±0.33		1.52 ± 0.7		14.08 ± 2.7	

PD: progressive disease, Non-PD: non progressive disease, naive: CD25^high^CD127^−/low^CD152^−^FoxP3^low^CD45RO^−^, effector: CD25^high^CD127^low^CD152^+^FoxP3^+^CD45RO^+^, terminal effector: CD25^high^CD127^−^CD152^+^FoxP3^+^CD45RO^+^.

(^*,**^PD compared non-PD; p < 0.05, 0.01, KS test).

**Table 3:** Univariate and multivariate analysis of PFS and median OS for NSCLC patients.

	Hazard ratio (95% CI)	P value
A. Univariate Analysis		
PFS		
Age (<65 vs ≥65)	1.637 (0.738–3.632)	0.225
Gender (Male vs Female)	1.171 (0.486–2.820)	0.725
Histology (Non-Squamous vs Squamous)	1.001 (0.413–2.427)	0.998
Naive Tregs (above vs below 95% of controls)	1.502 (0.609–3.704)	0.377
Effector Tregs (above vs below 95% of controls)	3.050 (1.104–8.429)	0.032
Terminal Effector Tregs (below vs above 95% of controls)	2.740 (1.050–7.151)	0.039
OS		
Age (<65 vs ≥65)	1.115 (0.472–2.638)	0.803
Gender (Male vs Female)	1.559 (0.645–3.771)	0.324
Histology (Non-Squamous vs Squamous)	1.216 (0.443–3.338)	0.704
Naive Tregs (above vs below 95% of controls)	2.476 (1.016–6.034)	0.046
Effector Tregs (above vs below 95% of controls)	3.357 (1.006–11.199)	0.049
Terminal Effector Tregs (below vs above 95% of controls)	2.881 (0.960–8.644)	0.059
B. Multivariate Analysis		
PFS		
Effector Tregs (above vs below 95% of controls)	2.050 (1.104–6.429)	0.065
Terminal Effector Tregs (below vs above 95% of controls)	3.466 (1.005–11.946)	0.049
OS		
Naive Tregs (above vs below 95% of controls)	8.632 (2.226–33.468)	0.002
Effector Tregs (above vs below 95% of controls)	5.638 (0.985–32270)	0.052
Terminal Effector Tregs (below vs above 95% of controls)	7.417 (1.055–52.151)	0.044

naive: CD25^high^CD127^−/low^CD152^−^FoxP3^low^CD45RO^−^, effector: CD25^high^CD127^low^CD152^+^FoxP3^+^CD45RO^+^, terminal effector: CD25^high^CD127^−^CD152^+^FoxP3^+^CD45RO^+^.
