# Supplementary material for: Prognostic value of circulating regulatory T cell subsets in untreated non-small cell lung cancer patients
Source: Sci Rep. 2016 Dec 15;6:39247. doi: 10.1038/srep39247 (PMC5157012; doi:10.1038/srep39247)

**Supplementary Information**

# Prognostic value of circulating regulatory T cell subsets in untreated non-small cell lung cancer patients

Athanasios Kotsakis[1,2,4], Filippos Koinis[1,2,4], Afroditi Katsarou[1,4], Marianthi Gioulbasani[1,4], Despoina Aggouraki[1,4], Nikolaos Kentepozidis[3,4], Vassilis Georgoulias[1,2,4] & Eleni-Kyriaki Vetsika[1,4]

[1]Laboratory of Translational Oncology, School of Medicine, University of Crete, GR-71110, Heraklion, Crete, Greece

[2]Department of Medical Oncology, University Hospital of Heraklion, GR-71110, Heraklion, Crete, Greece

[3]Department of Medical Oncology, 251 Air Force Hospital, Leof. P. Kanellopoulou, 115 25, Athens, Greece

[4]Hellenic Society of Immuno-Oncology, 55 Lombardou str., 11474, Athens, Greece

**Supplementary Table S1. Correlation between pathological characteristics and percentages of peripheral $CD3^+CD4^+CD25^+$ Tregs or $CD3^+CD4^+CD25^{high}$Tregs** (*, **, ***, **** NSCLC patients compared with healthy donors and brackets indicate comparison between histological types or pathological stage; p<0.05, 0.01, 0.001, 0.0001; KS test and unpaired T test)

| | **% of $CD3^+CD4^+CD25^+$ Mean ±SEM** | **P value** | | **% of $CD3^+CD4^+CD25^{high}$ Mean ±SEM** | **P value** | |
|---|---|---|---|---|---|---|
| Healthy Donor | 4.67 ± 1.5 | | | 1.23 ± 0.1 | | |
| **Histology** | | | | | | |
| Adenocarcinoma | 25.25±1.37**** | **0.002** | | 0.78±0.05*** | **0.001** | |
| | | | 0.9 | | | 0.56 |
| Squamous cell carcinoma | 24.64±1.69*** | **<0.0001** | 0.9 | 0.72±0.06*** | **0.001** | 0.54 |
| | | | 0.8 | | | 0.78 |
| Other types carcinoma | 23.72±2.68* | **0.007** | | 0.72±0.11** | **0.009** | |
| **Pathological Stage** | | | | | | |
| III | 21.79±2.27* | **0.01** | | 0.78±0.07** | **0.008** | |
| | | | 0.4 | | | 0.44 |
| IV | 25.47±1.09**** | **<0.0001** | | 0.74 ±0.04**** | **<0.0001** | |

**Supplementary Table S2. Correlation between pathological characteristics and percentages of peripheral $CD3^{+}CD4^{+}CD25^{+}FoxP3^{+}$ and $CD3^{+}CD4^{+}CD25^{high}FoxP3^{+}$ Tregs** (*, NSCLC patients compared with healthy donors and ‡‡ and brackets indicate comparison between histological types or pathological stage; p<0.05, 0.01; KS test and unpaired T test)

| | **% of $FoxP3^{+}$ $CD3^{+}CD4^{+}CD25^{+}$ Mean ±SEM** | **P value** | | | **% of $FoxP3^{+}$ $CD3^{+}CD4^{+}CD25^{high}$ Mean ±SEM** | **P value** | | |
|---|---|---|---|---|---|---|---|---|
| Healthy Donor | 35.46 ± 6.23 | | | | 54.81 ±5.9 | | | |
| **Histology** | | | | | | | | |
| Adenocarcinoma | 26.22±3.53* | **0.03** | | | 41.89±3.74 | 0.13 | | |
| | | | 0.4 | | | | ‡‡**0.006** | |
| Squamous cell carcinoma | 33.80±5.15 | 0.42 | | 0.3 | 57.44±4.83 | 0.89 | | 0.6 |
| | | | 0.9 | | | | 0.2 | |
| Other types carcinoma | 33.98±9.25 | 0.89 | | | 47.23±9.36 | 0.49 | | |
| **Pathological Stage** | | | | | | | | |
| III | 34.88±6.77 | 0.95 | | | 52.08±6.87 | 0.90 | | |
| | | | 0.5 | | | | 0.9 | |
| IV | 27.77±3.02 | 0.06 | | | 46.00±3.12 | 0.22 | | |

**Supplementary Table S3.** Relationship between pathological characteristics and percentages of peripheral $CD4^+$Tregs subtypes (*, ** NSCLC patients compared with healthy donors, ‡, ‡‡‡ and brackets indicate comparison between histological types or pathological stage; p<0.05, 0.01, 0.001, 0.0001; KS test and unpaired T test)

| **A** | **% of Naive $CD4^+$ Tregs Mean ±SEM** | **P value** | | |
|---|---|---|---|---|
| Healthy Donor | 1.33 ± 0.33 | | | |
| **Histology** | | | | |
| Adenocarcinoma | 1.22±0.26** | **0.006** | 0.11 | |
| Squamous cell carcinoma | 2.48±0.54 | 0.221 | | 0.64 |
| Other types carcinoma | 1.08±0.41 | 0.329 | 0.6 | |
| **Pathological Stage** | | | | |
| III | 2.26±0.62 | 0.161 | 0.068 | |
| IV | 1.45±0.25** | **0.01** | | |

| **B** | **% of Effector $CD4^+$ Tregs Mean ±SEM** | **P value** | | |
|---|---|---|---|---|
| Healthy Donor | 1.2 ± 0.61 | | | |
| **Histology** | | | | |
| Adenocarcinoma | 4.59±1.09 | 0.379 | 0.07 | |
| Squamous cell carcinoma | 4.56±1.63 | 0.169 | | 0.87 |
| Other types carcinoma | 1.30±0.87 | 0.978 | 0.34 | |
| **Pathological Stage** | | | | |
| III | 1.15±0.95 | 0.924 | ‡**0.03** | |
| IV | 5.04 ±0.97 | 0.096 | | |

| **C** | **% of Terminal Effector $CD4^+$ Tregs Mean ±SEM** | **P value** | | |
|---|---|---|---|---|
| Healthy Donor | 11.95 ± 3.74 | | | |
| **Histology** | | | | |
| Adenocarcinoma | 7.21±1.26 | 0.157 | ‡‡‡**0.001** | |
| Squamous cell carcinoma | 15.47±2.18 | 0.358 | | 0.12 |
| Other types carcinoma | 13.62±3.83 | 0.889 | 0.69 | |
| **Pathological Stage** | | | | |
| III | 12.57±2.55 | 0.379 | 0.16 | |
| IV | 9.72±1.23 | 0.606 | | |

**Supplementary Figure S1. Phenotypic analysis of (A) CD4$^{+}$ Tregs subpopulations and (B) subtypes in NSCLC patients.** Representative dot plots and histograms, as well as the gating strategy for identification and quantification of CD4$^{+}$ Tregs. Arrows indicate the sequence of gating. The gates for each dot plot and histogram are presented on the top of each box.

**A**

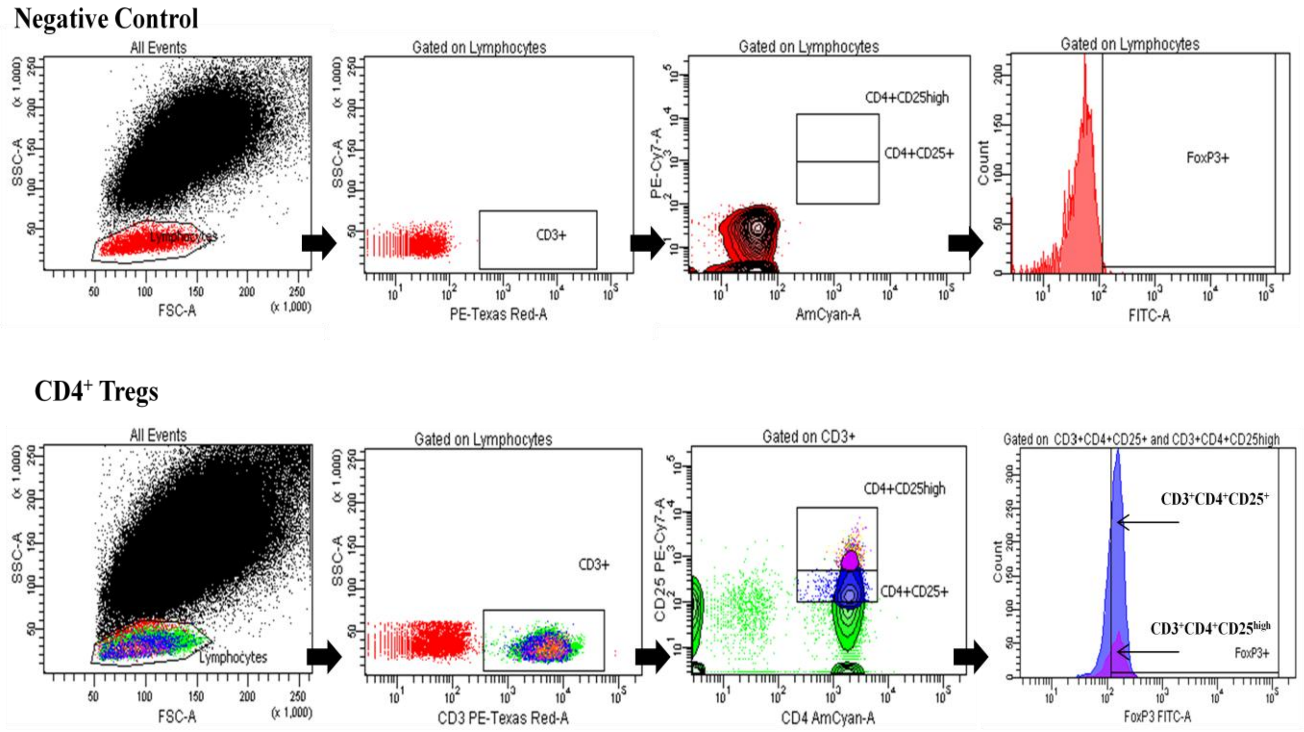

**B**

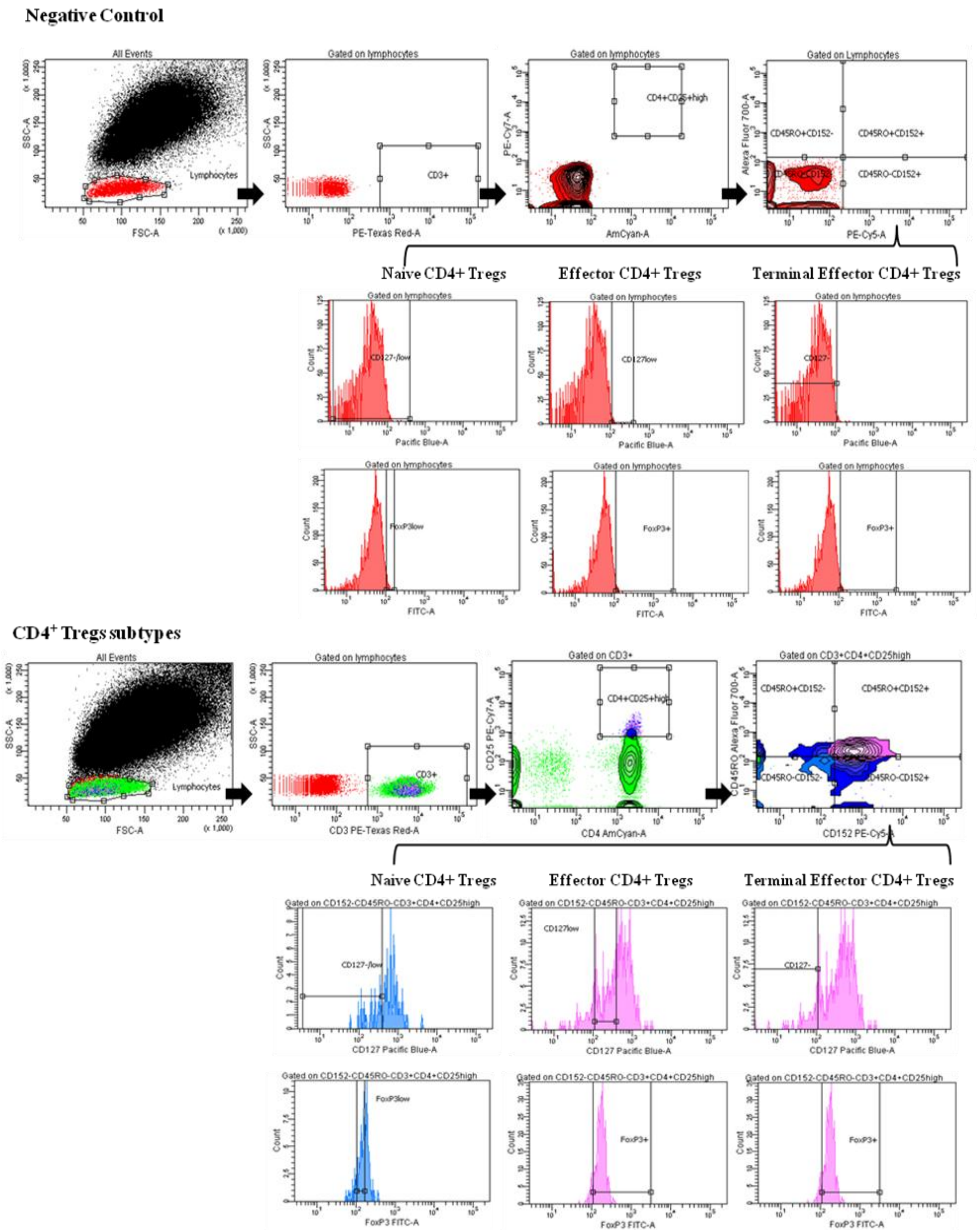

Negative Control
All Events
Lymphocytes
SSC-A
FSC-A
Gated on lymphocytes
CD3+
PE-Texas Red-A
CD4+CD25+high
PE-Cy7-A
AmCyan-A
Gated on Lymphocytes
CD45RO+CD152-
CD45RO+CD152+
CD45RO-CD152+
Alexa Fluor 700-A
PE-Cy5-A
Naive CD4+ Tregs
Effector CD4+ Tregs
Terminal Effector CD4+ Tregs
CD127-/low
CD127low
CD127-
Count
Pacific Blue-A
FoxP3low
FoxP3+
FITC-A
CD4+ Tregs subtypes
CD3 PE-Texas Red-A
Gated on CD3+
CD25 PE-Cy7-A
CD4 AmCyan-A
Gated on CD3+CD4+CD25high
CD45RO Alexa Fluor 700-A
CD45RO-CD152-
CD152 PE-Cy5-A
Gated on CD152-CD45RO-CD3+CD4+CD25high
CD127 Pacific Blue-A
FoxP3 FITC-A

**Supplementary Figure S2. Functionality of CD4$^+$ Treg subtypes in NSCLC patients**. Representative histograms of flow cytometry analysis of TGFβ and IL-10 gated in Naive, effector and Terminal effector Treg. The gates for histogram are presented on the top of each box. The positive expression of markers is compared to cells without Ab staining. Colors in histograms represent the different CD4$^+$ Treg subtypes; pink colour, naïve (CD25$^{high}$CD127$^{-/low}$CD152$^-$FoxP3$^{low}$CD45RO$^-$); bright green, effector (CD25$^{high}$CD127$^{low}$CD152$^+$FoxP3$^+$ CD45RO$^+$) and blue colour, terminal effector subtype (CD25$^{high}$CD127$^-$CD152$^+$ FoxP3$^+$CD45RO$^+$).

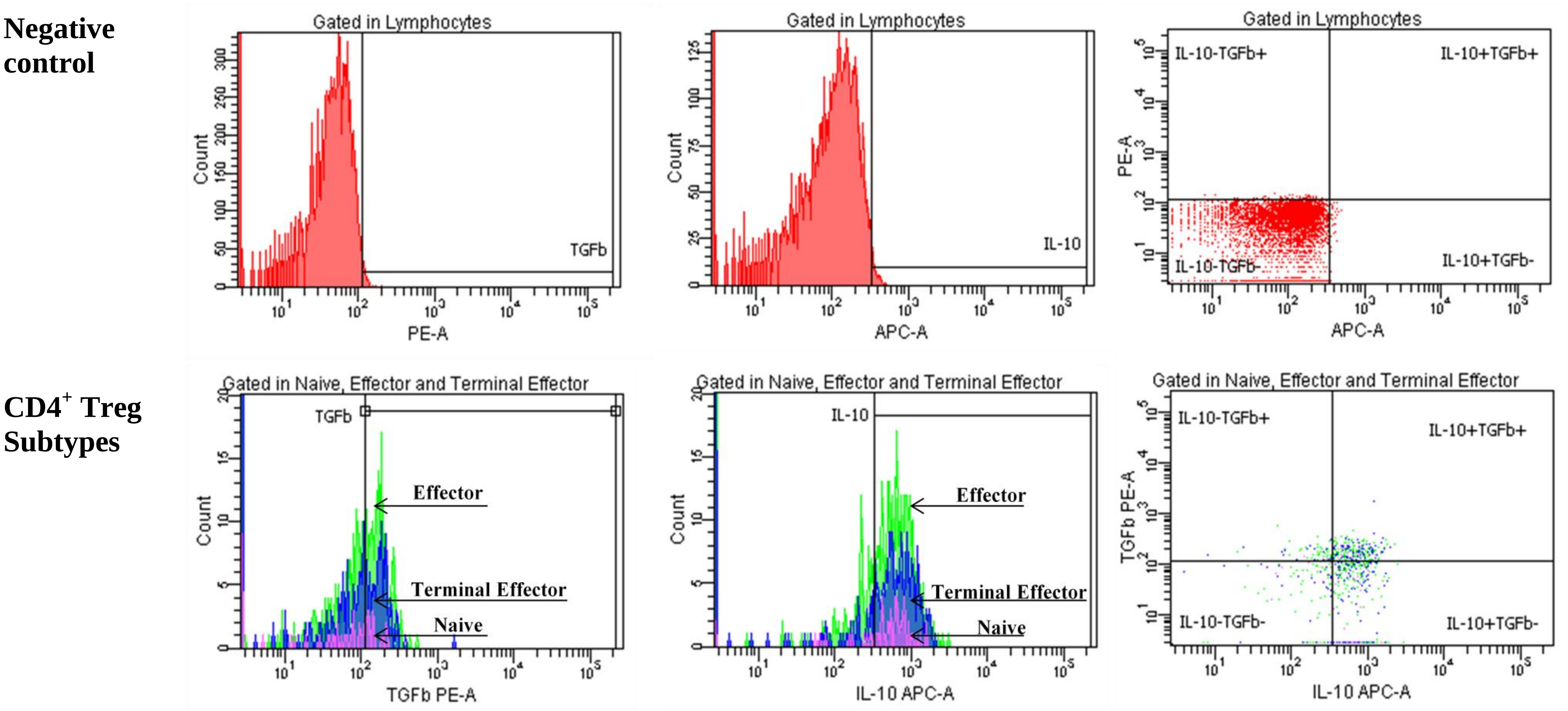

Supplement: Supplementary Information [file srep39247-s1.pdf]
